# Procalcitonin and Presepsin in Gram-Negative Sepsis: Diagnostic, Prognostic, and Antimicrobial Stewardship Perspectives

**DOI:** 10.7759/cureus.117204

**Published:** 2026-09-30

**Authors:** Bhaskar C Yadav, Harsha V Patil, Satish R Patil

**Affiliations:** 1 Department of Microbiology, Krishna Vishwa Vidyapeeth (Deemed to be University), Karad, IND

**Keywords:** antimicrobial stewardship, biomarkers, intensive care unit (icu), molecular diagnostics, multidrug-resistant gram-negative bacteria, presepsin, procalcitonin, sepsis

## Abstract

Because delayed pathogen identification and antibiotic susceptibility testing might delay effective treatment, sepsis caused by multidrug-resistant Gram-negative bacteria continues to be a significant problem in intensive care units. The clinical usefulness of procalcitonin (PCT) and presepsin as early indicators for Gram-negative sepsis diagnosis, disease severity prediction, treatment response monitoring, and antimicrobial stewardship is assessed in this review. While presepsin provides quick early identification and significant prognostic value, particularly in culture-negative sepsis, PCT is notably useful in distinguishing Gram-negative infections from Gram-positive or fungal etiologies. Before traditional culture findings are available, serial monitoring of both biomarkers may assist in detecting treatment failure, delayed biomarker clearance, and potential multidrug-resistant infections. Confounding variables like burns, trauma, renal failure, surgery, and sterile inflammation must be taken into account during interpretation, though. Rapid molecular diagnostics combined with PCT and presepsin may enhance early treatment choices, lower needless antibiotic exposure, and promote precision-based care for critically ill septic patients.

## Introduction and background

Sepsis is a life-threatening clinical condition associated with substantial morbidity and mortality and requires prompt recognition and appropriate antimicrobial therapy. Gram-negative bloodstream infections are an important cause of severe infection and bacteremia, and antimicrobial resistance among these pathogens can complicate treatment and adversely affect clinical outcomes [1-4]. The pathogenesis of Gram-negative bacteremia involves complex interactions between bacterial factors and the host immune response, which can contribute to systemic inflammation, endothelial dysfunction, coagulation abnormalities, and organ injury [3]. According to the Third International Consensus Definitions for Sepsis and Septic Shock (Sepsis-3), sepsis is defined as life-threatening organ dysfunction caused by a dysregulated host response to infection. Septic shock represents a severe subset of sepsis characterized by profound circulatory and metabolic abnormalities and is clinically identified by the requirement for vasopressors to maintain a mean arterial pressure of ≥65 mmHg and a serum lactate level >2 mmol/L despite adequate fluid resuscitation [4].

Sepsis is a major cause of ICU admission, prolonged hospital stays, increased healthcare costs, and mortality. Mortality remains high despite advances in supportive care and adherence to evidence-based treatment guidelines, particularly among patients with septic shock and multidrug-resistant (MDR) infections. Early detection and timely initiation of appropriate antimicrobial therapy remain essential for the successful management of sepsis. Because treatment delays are associated with increased mortality and progressive organ dysfunction, the Surviving Sepsis Campaign strongly recommends administering appropriate antimicrobial therapy as soon as possible [2,3].

Among the many pathogens responsible for sepsis, Gram-negative bacilli (GNB) remain particularly challenging. *Escherichia coli, Klebsiella pneumoniae, Pseudomonas aeruginosa, *and *Acinetobacter baumannii* are among the most frequent causes of bloodstream infections and septic shock associated with healthcare. These pathogens possess several virulence factors, including the ability to produce endotoxins, form biofilms, evade host immune responses, and acquire diverse antimicrobial resistance determinants [3].

The global emergence and spread of MDR Gram-negative organisms over the past two decades have made the treatment of serious infections considerably more challenging. The World Health Organization (WHO) has identified carbapenem-resistant Enterobacterales (CRE), carbapenem-resistant Acinetobacter baumannii (CRAB), and carbapenem-resistant Pseudomonas aeruginosa (CRPA) as high-priority pathogens requiring urgent research and the development of effective therapeutic strategies. Carbapenemases, such as Klebsiella pneumoniae carbapenemase (KPC), New Delhi metallo-β-lactamase (NDM), Verona integron-encoded metallo-β-lactamase (VIM), and OXA-type β-lactamases, are commonly identified in these organisms. These enzymes severely limit treatment options and contribute to increased morbidity and mortality [4].

One of the major challenges in contemporary clinical microbiology is the rapid detection of MDR infections. Conventional blood culture remains the reference standard for pathogen identification and antimicrobial susceptibility testing; however, culture-based techniques frequently require 24-72 hours or longer to yield conclusive results. False-negative cultures may result from fastidious organisms, a low bacterial burden, or prior antibiotic exposure, further delaying pathogen identification and targeted treatment. In the absence of conclusive microbiological data, clinicians are often required to initiate empirical broad-spectrum antimicrobial therapy during this critical period. Although empirical therapy is often necessary, prolonged use may lead to drug toxicity, antimicrobial resistance, alterations in the normal microbiota, and increased healthcare costs [4,5].

Considerable attention has been directed toward host-response biomarkers that can provide rapid diagnostic and prognostic information and help overcome these limitations. An ideal biomarker would facilitate the early detection of bacterial infections, differentiate infectious from non-infectious inflammatory conditions, predict disease severity, monitor treatment response, and potentially identify patients at risk of infection with MDR pathogens. Among the numerous biomarkers investigated, procalcitonin (PCT) and presepsin (soluble CD14 subtype; sCD14-ST) are two of the most promising candidates [5].

PCT is a well-established biomarker produced in response to systemic bacterial infection through cytokine-mediated pathways involving interleukin-1β, interleukin-6, and tumour necrosis factor-α. It has been extensively investigated for sepsis diagnosis, treatment monitoring, and antimicrobial stewardship. By contrast, presepsin is generated through activation of the innate immune response and is produced by the enzymatic cleavage of CD14 during bacterial phagocytosis. Because of its rapid release kinetics and close association with host-pathogen interactions, presepsin has attracted increasing interest as a potential biomarker for the early diagnosis and prognosis of sepsis [6,7].

Recent evidence suggests that serial monitoring of PCT and presepsin may provide clinically relevant information beyond the initial detection of infection. Changes in biomarker concentrations over time may provide additional information for assessing treatment response and ongoing inflammation. Therefore, these biomarkers may serve as useful adjuncts to clinical and microbiological assessment and may support antimicrobial stewardship, particularly in monitoring response to therapy and informing antibiotic de-escalation decisions [8].

Despite increasing interest in PCT and presepsin as biomarkers in sepsis, uncertainty remains regarding their ability to specifically identify Gram-negative infections and predict multidrug-resistant pathogens. Their potential role in monitoring treatment response and supporting antimicrobial stewardship also requires careful interpretation, particularly because these biomarkers cannot identify specific antimicrobial resistance mechanisms and presepsin concentrations may be influenced by renal dysfunction. Therefore, this review evaluates the biological basis, diagnostic and prognostic performance, treatment-response kinetics, limitations, and potential antimicrobial stewardship applications of PCT and presepsin in Gram-negative and multidrug-resistant sepsis, with particular emphasis on their complementary role alongside conventional microbiological and emerging rapid molecular diagnostic approaches [8].

Methods

Search Strategy

A literature search was conducted using PubMed/MEDLINE, Scopus, Web of Science, Embase, and Google Scholar to identify studies published between January 2000 and March 2026. Search terms included “sepsis”, “septic shock”, “Gram-negative sepsis”, “multidrug-resistant organisms”, “procalcitonin”, “PCT”, “presepsin”, “sCD14-ST”, “biomarkers”, “antimicrobial stewardship”, and “molecular diagnostics”, combined using Boolean operators (AND/OR).

English-language systematic reviews, meta-analyses, clinical trials, observational studies, and international guidelines evaluating the diagnostic, prognostic, and antimicrobial stewardship roles of PCT and/or presepsin in sepsis were included. Studies involving animal models, conference abstracts, commentaries, editorials, and articles lacking relevant clinical data were excluded. Additional articles were identified through manual screening of reference lists. The selected literature was narratively synthesised according to biomarker kinetics, diagnostic accuracy, prediction of multidrug-resistant pathogens, stewardship applications, and future diagnostic perspectives.

This review represents a narrative synthesis based on a structured literature search and predefined inclusion and exclusion criteria, without formal meta-analysis or statistical pooling. No formal meta-analysis, pooled effect-size calculations, or statistical significance testing was performed. Instead, the findings were qualitatively analysed and summarised based on the included studies. The literature selection process is illustrated in Figure 1.

**Figure 1:**
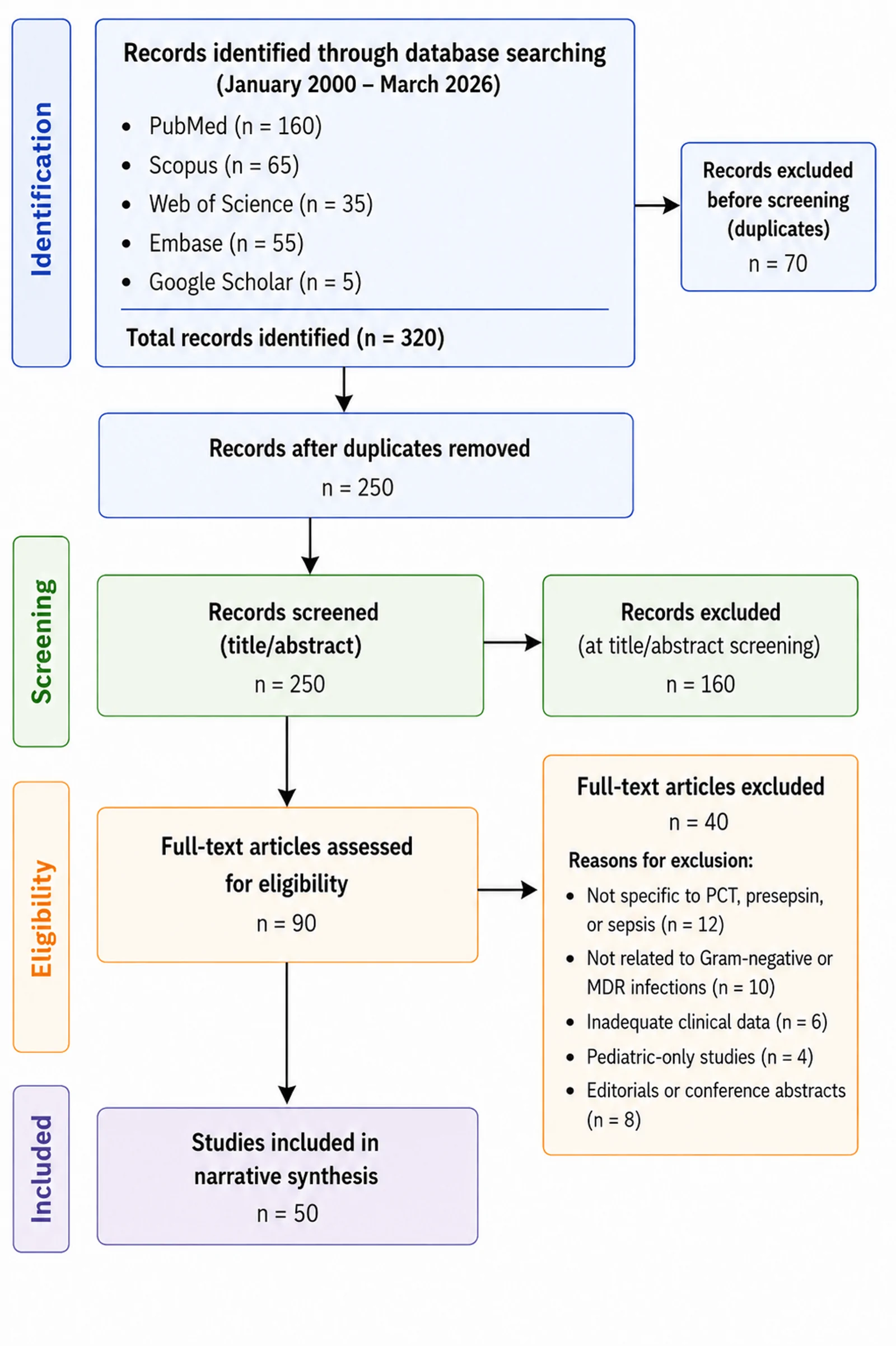
Preferred Reporting Items for Systematic Reviews and Meta-Analyses (PRISMA) flow diagram showing the identification, screening, eligibility assessment, and inclusion of studies evaluating procalcitonin and presepsin in multidrug-resistant Gram-negative sepsis.

Study Selection and Exclusion

Studies were initially screened based on their titles and abstracts, followed by full-text assessment of potentially eligible articles. A total of 160 records were excluded during the title and abstract screening stage. Of the articles assessed for full-text eligibility, 39 were excluded for the following reasons: 12 were not specific to PCT, presepsin, or sepsis; 10 were not related to Gram-negative or multidrug-resistant (MDR) infections; six did not provide adequate clinical data; four included only paediatric populations; and seven were editorials or conference abstracts. The remaining eligible studies were included in the narrative synthesis. The study selection process is summarized in Figure 1.

## Review

Understanding the kinetic behaviour of biomarkers is essential for their effective use in the diagnosis and management of severe Gram-negative sepsis. The clinical value, release mechanisms, response kinetics, and elimination patterns of PCT and presepsin are strongly influenced by their biological origins. These characteristics affect not only their diagnostic performance but also their utility in monitoring treatment response, predicting disease severity, and guiding antimicrobial stewardship decisions. Clinical and epidemiological factors, including nosocomial acquisition, underlying hematological malignancy, pulmonary infection, and previous exposure to antipseudomonal antibiotics, have been associated with an increased risk of multidrug-resistant (MDR) *Pseudomonas aeruginosa* bloodstream infection [7].

Mechanistic origins of procalcitonin (PCT)

PCT is a 116-amino acid precursor of the hormone calcitonin and is normally produced by the parafollicular C-cells of the thyroid gland. Under physiological conditions, PCT is intracellularly cleaved into calcitonin, resulting in very low or undetectable circulating concentrations in healthy individuals. However, during systemic bacterial infection, this tightly regulated physiological process undergoes substantial alteration. Inflammatory mediators, including interleukin-1β (IL-1β), interleukin-6 (IL-6), and tumour necrosis factor-α (TNF-α), stimulate widespread extra-thyroidal production of PCT by tissues, such as the liver, lungs, kidneys, adipose tissue, and peripheral blood mononuclear cells in response to microbial invasion [8,9].

PCT exhibits a relatively rapid kinetic profile and is more strongly associated with bacterial infections than conventional inflammatory markers, such as C-reactive protein (CRP). Following the onset of bacterial infection, serum PCT concentrations typically begin to increase within three to four hours, become markedly elevated within six to 12 hours, and reach peak concentrations between 12 and 24 hours. With a biological half-life of approximately 22-35 hours, PCT concentrations gradually decline once the bacterial stimulus is controlled [8,9].

The magnitude of PCT elevation is frequently associated with the severity of infection and bacterial burden. PCT concentrations are often substantially higher in patients with severe sepsis, septic shock, or Gram-negative bacteraemia than in those with localised infections or non-infectious inflammatory conditions. The high endotoxin burden associated with Gram-negative bacteria can trigger a stronger cytokine response and more pronounced PCT production. Consequently, PCT is a valuable biomarker for differentiating Presepsin is a biomarker of sepsis whose production is closely associated with CD14-mediated phagocytic processes during the host response to infection [9].

Serial monitoring of PCT concentrations can also provide valuable information regarding treatment response. A progressive decline in PCT levels following antimicrobial therapy generally suggests effective infection control, whereas persistently elevated or increasing concentrations may indicate treatment failure, inadequate source control, or infection caused by MDR organisms. Consequently, PCT has become one of the most extensively studied biomarkers for diagnostic and prognostic purposes in critical care medicine [9].

Mechanistic origins of presepsin (sCD14-ST)

Presepsin, also known as soluble CD14 subtype (sCD14-ST), is a relatively novel biomarker generated through interactions between invading microorganisms and cells of the innate immune system. The pattern-recognition receptor CD14 is primarily expressed on the surface of monocytes, macrophages, and neutrophils. It plays an important role in recognizing pathogen-associated molecular patterns, particularly lipopolysaccharide (LPS), the major endotoxin present in the outer membrane of Gram-negative bacteria [8,9].

During bacterial invasion, CD14 interacts with lipopolysaccharide-binding protein (LBP) and LPS, forming a receptor complex that initiates downstream inflammatory signalling pathways. Following bacterial phagocytosis, membrane-bound CD14 is enzymatically cleaved within phagolysosomes, releasing a soluble fragment known as presepsin. Because this process occurs directly during bacterial engulfment, presepsin production reflects active host-pathogen interaction rather than secondary cytokine amplification [9].

This distinctive biological mechanism may account for the rapid kinetics of presepsin. Serum concentrations may increase within approximately two hours of bacterial invasion, often earlier than those of PCT and several other inflammatory markers. Peak concentrations are generally observed during the early stages of systemic infection, making presepsin potentially useful for the early identification of sepsis. In addition, its relatively short half-life of approximately four to five hours may allow serum concentrations to respond rapidly to changes in infection status [8,9].

Numerous studies have demonstrated significant associations between elevated presepsin concentrations and sepsis severity, organ failure, and mortality. In contrast to PCT, which primarily reflects cytokine-mediated systemic inflammation, presepsin is considered a marker of innate immune activation and phagocytic activity. This distinction may contribute to its diagnostic utility in certain patient populations, particularly those with culture-negative sepsis or those who have received empirical antimicrobial therapy before microbiological sampling [9].

Why the Gram-negative endotoxin load matters

Owing to the distinct biological properties of bacterial endotoxin, both PCT and presepsin may be particularly useful in the assessment of Gram-negative infections. LPS, a major component of the outer membrane of Gram-negative bacteria, is a potent activator of the innate immune response. Recognition of circulating LPS can trigger inflammatory signaling, cytokine release, and endothelial activation, contributing to the pathophysiology of sepsis [10].

The high LPS burden associated with Gram-negative bacteria can strongly stimulate CD14-mediated immune responses and contribute to increased presepsin concentrations. Because LPS plays a central role in CD14 activation and bacterial phagocytosis, experimental studies have suggested that presepsin concentrations may be higher in response to Gram-negative pathogens than to Gram-positive infections [10,11].

Similarly, severe Gram-negative bacteraemia can induce substantial cytokine release and consequently increase PCT concentrations. Patients with Gram-negative bloodstream infections often exhibit higher PCT levels than those with Gram-positive or fungal infections because endotoxin is a potent inducer of IL-1β, IL-6, and TNF-α production. Several clinical studies have reported higher PCT concentrations in patients with Gram-negative bacteremia compared with those with Gram-positive bacteremia, suggesting that PCT may provide supportive information regarding the likely bacterial etiology [11].

Therefore, markedly elevated PCT concentrations, particularly when accompanied by elevated presepsin levels, may provide supportive evidence of Gram-negative infection and assist clinicians in selecting appropriate empirical antimicrobial therapy. In critically ill patients with suspected sepsis, the combined interpretation of these biomarkers may provide complementary information regarding host immune activation and the underlying infectious process, thereby supporting early clinical decision-making [11].

Diagnostic accuracy for Gram-negative pathogens

Assessing the diagnostic accuracy of PCT and presepsin in sepsis remains challenging because no single test provides a perfect reference standard. Although blood cultures remain the conventional reference method for pathogen identification, they are limited by prolonged turnaround times, false-negative results, and reduced sensitivity following prior antibiotic exposure. Several systematic reviews and meta-analyses have therefore evaluated the diagnostic performance of host-response biomarkers to improve early sepsis detection and support timely clinical decision-making [12].

The Evidence: Meta-Analyses and AUROC Comparisons

Systematic reviews have reported moderate-to-high diagnostic accuracy for presepsin in the detection of sepsis across diverse patient populations. Meta-analyses including prospective and retrospective studies have reported pooled sensitivities ranging from 76% to 82% and specificities ranging from 70% to 78%. Aggregate area under the receiver operating characteristic curve (AUROC) values have generally ranged from 0.80 to 0.89. In some studies, the diagnostic sensitivity of presepsin increased with disease severity, with values exceeding 90% among patients with septic shock [13].

PCT has demonstrated comparable diagnostic performance. Several pooled analyses have reported an overall AUROC of approximately 0.84 for sepsis diagnosis, with sensitivities of approximately 78% (95% CI: 74-81%) and specificities of approximately 77% (95% CI: 71-82%). Head-to-head comparisons between PCT and presepsin have frequently shown no statistically significant differences in overall sensitivity or specificity for the diagnosis of bacterial sepsis, indicating that both biomarkers may serve as useful adjuncts to clinical assessment and microbiological testing [13,14].

Diagnostic Accuracy for Gram-Negative Pathogens

Although both biomarkers demonstrate comparable performance in the diagnosis of sepsis overall, important differences may emerge when evaluating Gram-negative infections specifically. The strong endotoxin-mediated inflammatory response induced by Gram-negative organisms can substantially increase PCT production through enhanced cytokine release. Consequently, PCT may be useful in differentiating Gram-negative bacteremia from Gram-positive and fungal bloodstream infections, with significantly higher PCT concentrations reported in patients with Gram-negative bacteremia than in those with Gram-positive and fungal infections [15].

Several studies have reported significantly higher PCT concentrations in patients with confirmed Gram-negative bloodstream infections than in those with Gram-positive bacteraemia. In a large diagnostic cohort, PCT demonstrated an AUROC of approximately 0.765 for differentiating Gram-positive from Gram-negative infections, with a cut-off value of approximately 10.8 ng/mL providing useful discriminatory performance. PCT has also demonstrated good performance in differentiating Gram-negative bacteraemia from invasive fungal infections, with AUROC values approaching 0.94 in certain patient populations [16-19].

Additional evidence suggests that elevated PCT concentrations may be associated with bacterial DNAemia detected using advanced molecular techniques, such as metagenomic next-generation sequencing (mNGS). This finding further supports the potential role of PCT as a marker of substantial bacterial burden and severe systemic infection [16].

Comparative performance of presepsin

Presepsin primarily reflects phagocytosis-induced activation of the innate immune response rather than pathogen-specific endotoxin signalling. Consequently, it has limited specificity for identifying the precise pathogen class but may demonstrate high sensitivity for bacterial infection. Although presepsin concentrations increase substantially during both Gram-positive and Gram-negative infections, studies have reported lower AUROC values for specifically identifying Gram-negative pathogens, often ranging from 0.37 to 0.58 [17].

Despite these limitations in aetiological differentiation, presepsin may have important prognostic value. Several studies have demonstrated associations between elevated presepsin concentrations and adverse clinical outcomes, including septic shock, organ failure, and mortality. Meta-analyses evaluating prognostic performance have reported that elevated presepsin concentrations are independently associated with an increased risk of 28-day mortality, with odds ratios exceeding 3.0 in several cohorts [18,19].

Clinical interpretation and diagnostic implications

The available evidence suggests that PCT and presepsin should be regarded as complementary rather than competing biomarkers. In addition to providing greater discrimination of Gram-negative bacteraemia, PCT may help identify patients at increased risk of MDR Gram-negative infections. Presepsin, in contrast, may provide valuable prognostic information regarding disease severity and mortality risk while maintaining high sensitivity for early bacterial infection [20].

Therefore, the greatest clinical benefit may be achieved through the combined interpretation of both biomarkers. Elevated PCT concentrations may support the identification of likely Gram-negative aetiologies, whereas concurrent presepsin measurements may improve risk stratification, facilitate the early recognition of severe disease, and support timely therapeutic decision-making before definitive microbiological results become available [20,21].

PCT and Presepsin in MDR Gram-Negative Sepsis

The increasing prevalence of MDR Gram-negative bacteria in intensive care units has made sepsis management one of the most challenging aspects of modern critical care medicine. Although early recognition of sepsis remains essential, a major clinical concern is whether the causative organism is susceptible to conventional empirical therapy or possesses resistance mechanisms that may result in therapeutic failure. Carbapenem-resistant *Pseudomonas aeruginosa* (CRPA) and carbapenem-resistant *Acinetobacter baumannii* (CRAB) are associated with increased mortality, prolonged hospitalization, and higher healthcare costs. However, persistently elevated biomarker concentrations may reflect inadequate source control, high organism burden, delayed sampling, renal dysfunction, severe tissue injury, or ongoing inflammation and should not be interpreted as evidence of antimicrobial resistance alone. Furthermore, PCT and presepsin cannot identify specific resistance determinants, such as *Klebsiella pneumoniae* carbapenemase (KPC), New Delhi metallo-β-lactamase (NDM), Verona integron-encoded metallo-β-lactamase (VIM), or oxacillinase (OXA). Therefore, microbiological and susceptibility testing remains essential for confirming resistant pathogens and guiding definitive antimicrobial therapy [22,23].

Biomarker Kinetics and Treatment Response: Delayed Biomarker Clearance as a Marker of Therapeutic Failure

Although neither PCT nor presepsin directly identifies antimicrobial resistance genes, both biomarkers may provide valuable information regarding the effectiveness of antimicrobial therapy. Their potential role in predicting multidrug resistance is based on the premise that effective antimicrobial therapy should promote Effective bacterial clearance reduces inflammatory stimulation and may subsequently lead to declining biomarker concentrations [23].

Effective antimicrobial therapy is generally associated with declining biomarker concentrations as infection and systemic inflammation resolve. PCT typically decreases progressively during clinical recovery, while presepsin may show relatively rapid changes because of its short biological half-life [24].

Persistent or delayed decline of PCT and presepsin during treatment may be associated with ongoing infection, inadequate source control, ineffective antimicrobial therapy, or poor clinical response. Although such patterns may occur in infections caused by MDR organisms, they are not specific for antimicrobial resistance and should not be used alone to infer the presence of resistant pathogens [24-26].

MDR Gram-Negative Pathogens and Biomarker Dynamics

*Pseudomonas aeruginosa *and *Acinetobacter baumannii *are among the most challenging Gram-negative pathogens because of their extensive intrinsic and acquired resistance mechanisms. Treatment failure and prolonged infections may result from mechanisms such as efflux pump overexpression, porin loss, β-lactamase production, and biofilm formation [25].

Patients with infections caused by MDR non-fermenting Gram-negative bacteria may exhibit delayed biomarker clearance and persistently elevated biomarker concentrations. Studies evaluating bloodstream infections caused by resistant Gram-negative pathogens have reported associations between persistently elevated PCT concentrations and microbiological treatment failure. Similarly, sustained elevation of presepsin concentrations during the first week of treatment has been associated with prolonged infection and ineffective antimicrobial therapy [25,26].

In one study evaluating MDR Gram-negative bacteraemia, PCT concentrations above approximately 11 ng/mL were strongly associated with resistant infections. Similarly, failure of PCT concentrations to decline following therapy has been identified as an independent predictor of mortality among patients with resistant Gram-negative infections [26].

Carbapenemase-Producing Organisms and Persistent Biomarker Elevation

The emergence of carbapenemase-producing Gram-negative bacteria has further complicated the management of sepsis. Enzymes such as KPC-, NDM-, VIM-, and OXA-type carbapenemases can hydrolyse carbapenem antibiotics, thereby limiting the effectiveness of conventional treatment regimens [26].

When infections caused by carbapenemase-producing organisms are treated with ineffective empirical therapy, bacterial growth may persist despite antibiotic exposure. Clinically, the resulting prolonged inflammatory activation may manifest as persistently elevated PCT and presepsin concentrations. Studies have reported associations between persistently elevated presepsin concentrations after day seven, failure of PCT normalisation, ineffective antimicrobial therapy, and unfavourable clinical outcomes [26].

Importantly, these biomarker patterns may emerge several days before the underlying resistance mechanism is identified through conventional susceptibility testing. Consequently, serial monitoring of biomarker kinetics may provide an early physiological indication of persistent infection, possible antimicrobial resistance, and impending therapeutic failure [27].

Clinical implications

In the context of MDR prediction, PCT and presepsin are most useful for identifying patients whose infections are not responding adequately to empirical therapy rather than for directly diagnosing antimicrobial resistance. Persistently elevated biomarker concentrations, delayed clearance, or increasing levels despite appropriate supportive care should prompt clinicians to reassess antimicrobial coverage, evaluate source control, and consider the possibility of MDR infection [28].

Therefore, when clinically indicated, serial biomarker monitoring may serve as an early warning tool to complement microbiological testing and support timely modification of antimicrobial therapy. By providing real-time information regarding host-pathogen interactions and treatment response, PCT and presepsin may provide complementary information that could support antimicrobial stewardship in critically ill patients with suspected MDR Gram-negative sepsis [29].

Clinical Application: Biomarker-Guided Stewardship

Beyond their diagnostic utility, the integration of PCT and presepsin into routine intensive care practice has become an important component of contemporary antimicrobial stewardship programs (ASPs). Antimicrobial stewardship aims to optimise antibiotic use, improve patient outcomes, minimise adverse drug events and healthcare costs, and slow the emergence of antimicrobial resistance. Effective stewardship is particularly important in intensive care units (ICUs), where MDR Gram-negative bacteria are common and exposure to broad-spectrum antibiotics is widespread [29,30].

De-escalation Protocols and Safe Antibiotic Discontinuation

Among biomarkers used to guide antimicrobial therapy, PCT has the most extensive evidence supporting its use in determining antibiotic duration and discontinuation. Several randomised controlled trials and meta-analyses have demonstrated that serial PCT measurements can reduce antibiotic exposure without increasing mortality, treatment failure, or recurrent infection [30].

Most PCT-guided protocols recommend considering antibiotic discontinuation when serum concentrations decrease by at least 80% from baseline or fall below predefined thresholds, typically between 0.25 and 0.5 ng/mL. Studies using PCT-guided algorithms have consistently demonstrated reductions in antibiotic treatment duration of approximately two to four days. Importantly, these reductions may decrease unnecessary antibiotic exposure and selective pressure for resistant organisms without compromising clinical outcomes [31].

Point-of-care PCT testing may further strengthen antimicrobial stewardship by providing rapid information to support timely clinical decision-making. Early recognition of treatment response may allow clinicians to discontinue unnecessary antibiotics sooner, thereby reducing drug-related toxicity while preserving the effectiveness of broad-spectrum antimicrobial agents [32].

Emerging role of presepsin in antibiotic stewardship

Although the evidence supporting presepsin-guided stewardship is less extensive than that for PCT, recent studies have reported encouraging findings. Because presepsin concentrations increase rapidly during infection and may decline quickly following successful treatment, serial monitoring may provide an early indication of therapeutic response or failure [33].

Several studies suggest that presepsin-guided management strategies may reduce antibiotic exposure while maintaining favourable clinical outcomes. Comparative studies have reported that patients managed using presepsin-based algorithms experienced more antibiotic-free days than those managed according to conventional clinical criteria alone [33].

Presepsin may also be particularly useful in culture-negative sepsis, which is common among critically ill patients who receive empirical antimicrobial therapy before microbiological samples are collected. Because presepsin reflects ongoing phagocytic activity rather than direct pathogen detection, it may retain diagnostic utility even when blood cultures become negative following antibiotic exposure [34].

Biomarkers in Treatment Response and Antimicrobial Stewardship

Although antibiotic de-escalation is an important stewardship objective, timely escalation of therapy for patients who fail to respond to empirical treatment is equally important. Serial biomarker measurements may help identify patients at increased risk of MDR infection and provide useful information regarding treatment response [35].

Persistently elevated or increasing PCT and presepsin concentrations may indicate ongoing infection, inadequate source control, or an unfavourable treatment response. Such findings should prompt reassessment of the patient's clinical condition and microbiological data, including consideration of antimicrobial resistance when supported by the overall clinical context. Biomarker results alone should not be used to justify antimicrobial escalation [35].

Stewardship and Novel Reserve Antibiotics

Serial biomarker monitoring may provide supplementary information on treatment response in patients with MDR Gram-negative sepsis. However, persistent biomarker elevation cannot distinguish resistant from susceptible infections or identify specific resistance mechanisms. Interpretation should therefore be integrated with microbiological testing, antimicrobial susceptibility results, source control, and clinical response [36].

Studies evaluating ceftazidime-avibactam therapy have reported associations between serial PCT measurements, treatment response, and clinical outcomes. Therefore, biomarker monitoring may serve as an additional tool for assessing the response to newer antimicrobial agents in critically ill patients with MDR infections [36].

Future Directions in Stewardship

Future antimicrobial stewardship strategies will likely incorporate integrated approaches combining real-time microbiological surveillance, artificial intelligence-supported clinical decision systems, rapid molecular diagnostics, and host-response biomarkers. By combining pathogen identification with serial monitoring of host inflammatory responses, clinicians may be able to make more informed treatment decisions and reduce unnecessary antibiotic exposure. Accordingly, PCT and presepsin may be regarded not only as diagnostic biomarkers but also as dynamic tools that support individualised antimicrobial management throughout the course of critical illness. Their integration into evidence-based stewardship strategies may represent an important step towards precision medicine in sepsis management [36].

Confounding variables in the ICU

Although PCT and presepsin have important diagnostic and prognostic value, neither biomarker should be interpreted in isolation. Critically ill patients frequently experience complex physiological disturbances that may alter biomarker concentrations independently of infection. Important confounding factors include acute kidney injury (AKI), chronic renal dysfunction, renal replacement therapy, major surgery, severe trauma, and burn injury. Understanding the effects of these factors is essential for the accurate interpretation of biomarker kinetics in intensive care settings [37].

Renal Dysfunction and Biomarker Clearance

Acute kidney injury is a common complication of severe sepsis and septic shock. Because PCT and presepsin are low-molecular-weight molecules that are partially cleared by the kidneys, impaired renal function may substantially affect their circulating concentrations [38].

Presepsin appears to be more strongly affected by renal dysfunction than PCT. Several studies have reported a strong inverse relationship between serum presepsin concentrations and estimated glomerular filtration rate (eGFR). Even in the absence of infection, baseline presepsin concentrations may increase as renal function declines, thereby reducing diagnostic specificity. Patients with advanced chronic kidney disease or those undergoing haemodialysis may have substantially elevated presepsin concentrations that overlap with values commonly observed in sepsis [39].

To address this limitation, several investigators have proposed renal function-adjusted diagnostic thresholds. Available evidence suggests that individuals with preserved renal function (eGFR ≥60 mL/min/1.73 m²) may benefit from a presepsin cut-off of approximately 500 pg/mL, whereas patients with severe renal impairment may require thresholds of approximately 1000 pg/mL or higher. Such adjustments may reduce false-positive interpretations and improve diagnostic accuracy [39].

Acute Kidney Injury and PCT Interpretation

Although PCT appears to be less affected by renal dysfunction than presepsin, severe AKI can still alter its kinetics. Reduced renal clearance may contribute to persistently elevated PCT concentrations even when infection is effectively controlled. In addition, critically ill patients receiving continuous renal replacement therapy (CRRT) may exhibit altered biomarker elimination patterns, further complicating interpretation [40].

Therefore, clinicians should exercise caution when applying conventional PCT-guided stewardship algorithms in patients with severe renal dysfunction. Failure to achieve the expected decline in PCT concentrations may reflect impaired biomarker clearance rather than treatment failure. Interpretation should therefore incorporate clinical findings, microbiological results, and trends in organ function [40].

Sterile Inflammation and Systemic Inflammatory Response Syndrome

Distinguishing sepsis from non-infectious systemic inflammatory response syndrome (SIRS) remains a major challenge in critical care medicine. Patients may develop severe inflammatory responses that resemble bacterial sepsis following major surgery, severe trauma, burns, or cardiopulmonary bypass [41].

These sterile inflammatory conditions can induce cytokine production and activation of innate immune pathways, resulting in elevated concentrations of several commonly used inflammatory biomarkers. Significant increases in PCT may occur following extensive tissue injury because systemic cytokine activation can strongly influence PCT synthesis, even in the absence of infection. This may reduce diagnostic specificity and increase the risk of unnecessary antibiotic administration [41].

Advantages of presepsin in sterile inflammation

Presepsin appears to be less susceptible than PCT to elevation caused by sterile inflammatory stimuli. Its production is more closely associated with active phagocytosis and host-pathogen interactions than with widespread cytokine release. Consequently, several studies have suggested that presepsin may be useful in differentiating sterile SIRS from bacterial sepsis, particularly in patients with severe trauma or burns [42].

In patients with severe burns, presepsin has demonstrated better diagnostic performance than conventional inflammatory markers in some studies, particularly when extensive tissue damage produces marked inflammatory responses. In addition, presepsin may retain diagnostic value in culture-negative sepsis, which frequently occurs in patients who receive empirical antimicrobial therapy before microbiological sampling [43].

Trauma, Burns, and Culture-Negative Sepsis

Trauma and burn injuries represent distinct clinical settings characterised by extensive tissue damage, immune dysregulation, and frequent exposure to broad-spectrum antimicrobial therapy. Culture-negative sepsis is common in these settings because early antibiotic administration may suppress bacterial growth in blood cultures without completely eradicating the underlying infection. Studies evaluating biomarker performance in patients with burns have suggested that presepsin may be less affected by sterile inflammatory responses while remaining elevated during active infection. In contrast, PCT concentrations may increase substantially following severe trauma or burns even in the absence of bacterial infection [44].

Furthermore, kinetic studies following severe trauma have demonstrated associations between presepsin concentrations and subsequent infectious complications, potentially providing earlier diagnostic information than conventional inflammatory markers. These findings support the potential role of presepsin as a useful adjunct in critically ill patients whose complex inflammatory physiology limits the reliability of conventional diagnostic approaches [45].

Clinical Implications

The interpretation of PCT and presepsin in intensive care unit patients requires careful consideration of underlying physiological conditions. Renal failure, renal replacement therapy, major surgery, severe trauma, and burn injury can substantially influence biomarker concentrations. Failure to recognise these confounding factors may lead to diagnostic misinterpretation [45].

Among these confounding factors, renal dysfunction appears to have a greater effect on presepsin concentrations, necessitating consideration of renal function when interpreting diagnostic thresholds. In contrast, PCT may be more susceptible to elevation caused by sterile inflammation, potentially reducing its specificity in trauma and postoperative populations [46].

Therefore, optimal clinical use of these biomarkers requires integration with the individual patient's clinical context, laboratory findings, microbiological data, and serial kinetic patterns. When interpreted appropriately, PCT and presepsin may remain valuable tools for diagnosis, prognosis, and antimicrobial stewardship despite the complex physiological challenges encountered in critical care medicine [46].

Future perspectives

Future Perspectives: Toward Precision Sepsis Diagnostics

The integration of host-response biomarkers with advanced molecular diagnostic tools is becoming increasingly important in sepsis management. Conventional microbiological methods remain the reference standard for definitive pathogen identification and antimicrobial susceptibility testing; however, prolonged turnaround times may create a substantial delay between clinical presentation and the initiation of targeted therapy. Presepsin has demonstrated potential as a diagnostic and prognostic biomarker in sepsis; however, current evidence does not support its use as an independent predictor of antimicrobial resistance or as a basis for antibiotic escalation. Furthermore, presepsin concentrations are influenced by renal function, which may limit their interpretation in critically ill patients, particularly those with renal impairment. Therefore, future studies should evaluate presepsin alongside clinical assessment, microbiological findings, antimicrobial susceptibility testing, and rapid molecular diagnostics to determine its appropriate role in precision sepsis management and antimicrobial stewardship. [47].

Recent advances in molecular microbiology have enabled the development of rapid diagnostic platforms capable of identifying pathogens and resistance determinants directly from clinical specimens within hours. Multiplex polymerase chain reaction (PCR) assays, syndromic diagnostic panels, metagenomic sequencing technologies, and other molecular approaches can rapidly detect important resistance genes, including KPC-, NDM-, VIM-, IMP-, and OXA-type carbapenemases. These technologies may provide clinicians with early information regarding the likely causative pathogen and its resistance profile before conventional culture results become available [48].

However, molecular diagnostics alone cannot provide a comprehensive assessment of disease severity or treatment response. Although these technologies can identify pathogens and their genetic resistance determinants, they do not directly reflect the host's physiological response to infection. This limitation highlights the continuing value of biomarkers such as presepsin and PCT. Serial biomarker measurements may provide real-time information regarding inflammatory activity, treatment response, disease progression, and prognosis [49].

One promising approach to precision medicine in severe Gram-negative sepsis is the integration of rapid molecular diagnostics with host-response biomarkers. Molecular testing may identify pathogens and resistance mechanisms, while biomarker kinetics can simultaneously provide information regarding treatment response and potential clinical outcomes. Such integrated diagnostic strategies may help shorten the time to pathogen identification and antimicrobial susceptibility results, potentially enabling earlier optimization of antimicrobial therapy in critically ill patients [50].

## Conclusions

PCT and presepsin are complementary biomarkers with potential clinical value in the diagnosis and management of MDR Gram-negative sepsis. PCT may be particularly useful for differentiating Gram-negative bacterial infections from Gram-positive and fungal infections, monitoring treatment response, and supporting antimicrobial decision-making. Presepsin, in contrast, may provide valuable information regarding early bacterial infection, disease severity, and prognosis and may retain diagnostic utility in culture-negative sepsis.

Although neither biomarker directly detects antimicrobial resistance mechanisms, serial changes in their concentrations may provide useful information regarding treatment response and persistent infection. Persistently elevated or increasing biomarker concentrations, or inadequate clearance following antimicrobial therapy, may prompt clinicians to reassess antimicrobial coverage, source control, and the possibility of MDR infection before definitive susceptibility results become available. When interpreted alongside clinical findings, microbiological investigations, antimicrobial stewardship strategies, and rapid molecular diagnostic tools, PCT and presepsin may support earlier optimisation of antimicrobial therapy and more individualised management of patients with severe MDR Gram-negative sepsis.
